# Leicester Saturday Hospital Society

**Published:** 1918-02-23

**Authors:** 


### Leicester Saturday Hospital Society.

A RECORD YEAR.

At the recent anniversary meeting of the Melton Mowbray Branch of the Leicester Saturday Hospital Society record contributions were reported. The hon. secretary, Mr. J. Sutherland, in announcing this fact, stated that the local Society had 1,100 weekly contributors of a penny or a halfpenny, who, besides creating a local record, had helped to establish the record which the parent Society had announced—a contribution of £17,500. No better investment could be found for workpeople than a weekly contribution to the Leicester Royal Infirmary and the Saturday Hospital Society. The former institution had received eighty-one in-patients from Melton Mowbray during the year, and seventy-seven were given out-patient treatment, many of whom had spoken to him in terms of high praise of the attention and treatment accorded. Three members had received convalescent treatment in the parent Society's Homes. He referred to the memorial to perpetuate the memory of Sir Edward Wood, the founder and president of the parent Society, and intimated that the local branch would take its share in the scheme of assistance which the parent Society had in contemplation.

Mr. R. W. Brownlow, the hon. treasurer, expressed his satisfaction that a sum of not less than £240 would be remitted to the parent Society. The report was adopted, and the annual election of officers followed.

At Hinckley an arrangement exists between the Saturday Hospital Society and the local hospital committee under which the organising secretary of the Saturday Hospital Society (Mr. H. H. Woolley, J.P.) receives encouragement in the organising of systematic weekly contributions in the factories of the town on a sharing basis between the institutions, local contributors receiving all the advantages of membership of the Society. This arrangement has resulted in increased contributions. It seems very desirable that a similar system should be adopted in other centres of the county. Organisation is power, and with local committees administering one fund, to which all contributions are sent, increased giving is encouraged and great advantages afforded to local contributors, who, in addition, secure direct representation on the parent Society.

